# Dielectric behavior of ceramic–graphene composites around the percolation threshold

**DOI:** 10.1186/s11671-015-0921-4

**Published:** 2015-05-13

**Authors:** Lucía Fernández-García, Marta Suárez, José Luis Menéndez, Carlos Pecharromán, Rosa Menéndez, Ricardo Santamaría

**Affiliations:** Centro de Investigación en Nanomateriales y Nanotecnología, Consejo superior de Investigaciones Científicas (CSIC)—Universidad de Oviedo (UO)—Principado de Asturias, Avenida de la Vega 4-6, El Entrego, 33940 San Martin del Rey Aurelio (Asturias) Spain; Instituto de Ciencia de Materiales de Madrid, ICMM-CSIC, Madrid, Spain; Instituto Nacional del Carbón, INCAR-CSIC, Apartado 73, 33080 Oviedo, Spain

**Keywords:** Ceramic composites, Dielectric properties, Percolation threshold

## Abstract

Al_2_O_3_/graphene and BaTiO_3_/graphene composites with different concentrations of the conductive second phase, both below and above the percolation threshold, were prepared by the traditional ceramic processing route followed by spark plasma sintering. It is shown that the addition of graphene pins the grain growth of the ceramic matrix grains, leading to a change of the microstructure at low filler concentrations. As a consequence, the composites exhibit two percolation thresholds and their dielectric properties are not only determined by the dielectric properties of the constituents and their relative fractions but also the microstructure of the composite must be considered. Additionally, a giant increase of the dielectric constant has been found around the percolation thresholds in barium titanate–graphene composites. In particular, values of the dielectric constant up to 45,000 and 15,000 were found at 1 kHz in composites containing 0.4 and 0.6 wt. % graphene, respectively.

## Background

It is increasingly being recognized that new applications for materials require functions and properties that are not achievable with monolithic materials. In this sense, composites formed by a ceramic matrix and a conductive second phase are very interesting materials since their physical properties, such as optical, electrical, and magnetic properties as well as tribological, corrosion resistance, and wear properties can be tailored, making them attractive for many new applications [1–4]. In particular, the development of ceramic matrix–nanocarbon composites arises as a solution for applications in which materials with an electrical conductivity similar to those of metals and mechanical properties, particularly hardness, are simultaneously required [5].

The dielectric properties of insulator–conductor materials, such as conductivity and permittivity, show a critical behavior when the fraction of the conductive phase reaches the percolation threshold [6, 7], which has been the subject of interest of many studies [8–10]. It has been shown both experimentally and theoretically that the percolation threshold strongly depends on the aspect (length-to-diameter) ratio of the conductive particles [11–13]. Hence, it is not surprising that a number of experimental studies have verified the potential of carbon nanotubes as conductive fillers resulting in very low percolation thresholds [14, 15]. More recently, and due to their extraordinary properties, graphene is being incorporated to new conductor/insulator heterogeneous systems [16, 17]. In this regard, alumina and barium titanate–graphene composites have received a considerable interest in recent times for both their mechanical [18, 19] and electrical properties [20, 21]. In this work, we report how the dielectric properties of ceramic matrix composites are determined by the changes in the microstructure due to the introduction of a conductive second phase of graphene. More specifically, the dielectric properties of spark plasma sintered Al_2_O_3_/graphene composites with graphene concentrations between 0.56 and 1.24 % weight and BaTiO_3_/graphene composites with graphene concentrations between 0.1 and 0.6 % weight are studied in the frequency range of 10^−1^–10^6^ Hz.

## Methods

### Preparation of Graphene Oxide

Graphene oxide was synthesized from synthetic graphite by the modified Hummers method [22]. This method employs Hummers reagents with small amounts of NaNO_3_ and KMnO_4_. Concentrated H_2_SO_4_ (360 mL) was added to a mixture of synthetic graphite (7.5 g) and NaNO_3_ (7.5 g), and the mixture was cooled down to 0 °C using an ice bath. Afterwards, KMnO_4_ (45 g) was slowly introduced in small doses to keep the reaction temperature below 20 °C. The solution was heated to 35 °C and stirred for 3 h. At that point, a hydrogen peroxide (H_2_O_2_) 3 % solution (1.5 L) was slowly poured, giving rise to a pronounced exothermal effect up to 98 °C. The reaction mixture was stirred for 30 min and centrifuged (3700 rpm for 30 min) to discard the supernatant. The remaining solid material was then washed with 600 mL of water and centrifuged again; this process being repeated until the pH was neutral.

A colloidal suspension of individual graphene oxide sheets in purified water (1 mg·mL^−1^) was prepared in 1-L batches and kept under ultrasound for 10 h. Afterwards, the suspension was centrifuged (3700 rpm for 30 min) to discard the filtered supernatant. To prepare the suspensions for organic solvents, water was evaporated in a rotary evaporator and 10 mL of each solvent was dispersed into 1 mg of solid to then sonicate for 30 min. Details on the characteristics of graphene oxide are given elsewhere [22].

### Preparation of Al_2_O_3_/Graphene and BaTiO_3_/Graphene Composites

Al_2_O_3_/graphene powders with graphene concentrations from 0.56 to 1.24 % weight and BaTiO_3_/graphene powders between 0.1 and 0.6 % were prepared as follows: Al_2_O_3_ (Taimei TM-DAR, >99.99 % purity) and BaTiO_3_ (Inframat, >99.95 % purity) powders for ceramic matrices with an average particle size of around 150 and 700 nm, respectively, were dispersed in distilled water then mixed with the graphene oxide dispersion and finally ball milled for 1 h. The homogeneous mixtures were spray dried and uniaxially pressed (30 MPa) prior to spark plasma sintering in an FCT-HP D25/1 apparatus with a heating rate of 50 °C·min^−1^ under an applied pressure of 80 MPa, a holding time of 1 min and in vacuum (10^−1^ mbar). The final sintering temperature was 1500 °C in the case of Al_2_O_3_/graphene composites and 1100 °C for BaTiO_3_/graphene. Densities close to 100 % were found in all composites.

Powder X-ray diffraction analysis (D8 Advance, BRUKER) was used to determine possible high-temperature chemical reactions between components in sintered samples. Raman spectroscopy was performed on a Renishaw 2000 Confocal Raman Microprobe (Rhenishaw Instruments, England) using a 514.5-nm argon ion laser. Spectra were recorded from 1100 to 3500 cm^−1^. Microstructure was characterized by field emission scanning electron microscopy (FESEM) on a QUANTA FEG 650 and by transmission electron microscopy (TEM) on a JEOL-JEM 2100F. The dielectric properties of the samples were studied by standard low-frequency impedance measurements (PSM1735-NumetriQ).

## Results and Discussion

### Al_2_O_3_/Graphene

**Fig. 1:**
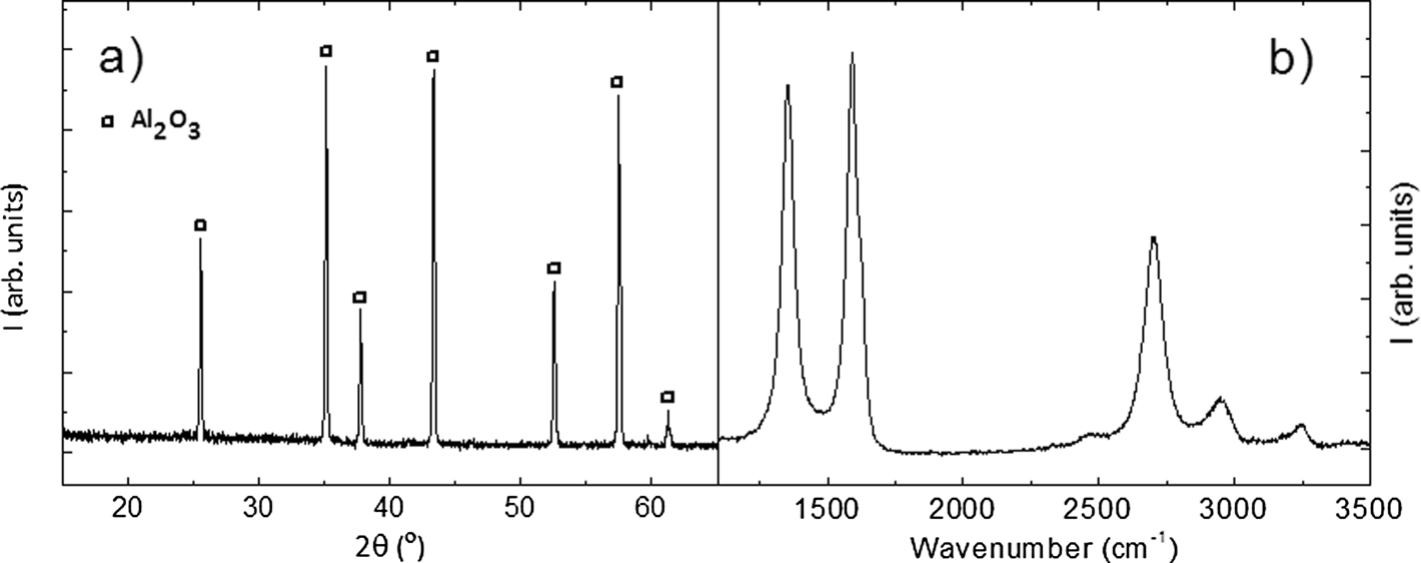
X-ray diffractogram (**a**) and Raman spectra (**b**) for the sample with the highest (1.24 % weight) graphene content

X-ray diffraction analysis of Al_2_O_3_/graphene composites (Fig. 1a) only shows the diffraction peaks corresponding to alumina, indicating that no reaction between the raw powders or decomposition of the matrix has taken place. In order to study the structure of the graphene after sintering, Raman spectra were taken (Fig. 1b). Bands *D* (1355 cm^−1^) and *G* (1583 cm^−1^) are narrow and present similar intensities (*I*_D_/*I*_G_ = 0.92). A shoulder corresponding to the *D’* band (1620 cm^−1^) is observed next to the G band and a well-defined structure is also present in the range 2500–3500 cm^−1^. A single and narrow 2*D* peak is centered around 2700 cm^−1^; therefore, according to Ferrari et al. [23], the predominant structure in the composite is one single-graphene layer. A qualitative comparison with the results by Botas et al. [22], considering the relative intensities of the *D* and *G* bands together with the shape of the 2*D* band, indicates that the state of the graphene in these composites is intermediate between those treated at 1000 and 2000 °C in that work, which agrees with the sintering temperature of 1500 °C.

**Fig. 2:**
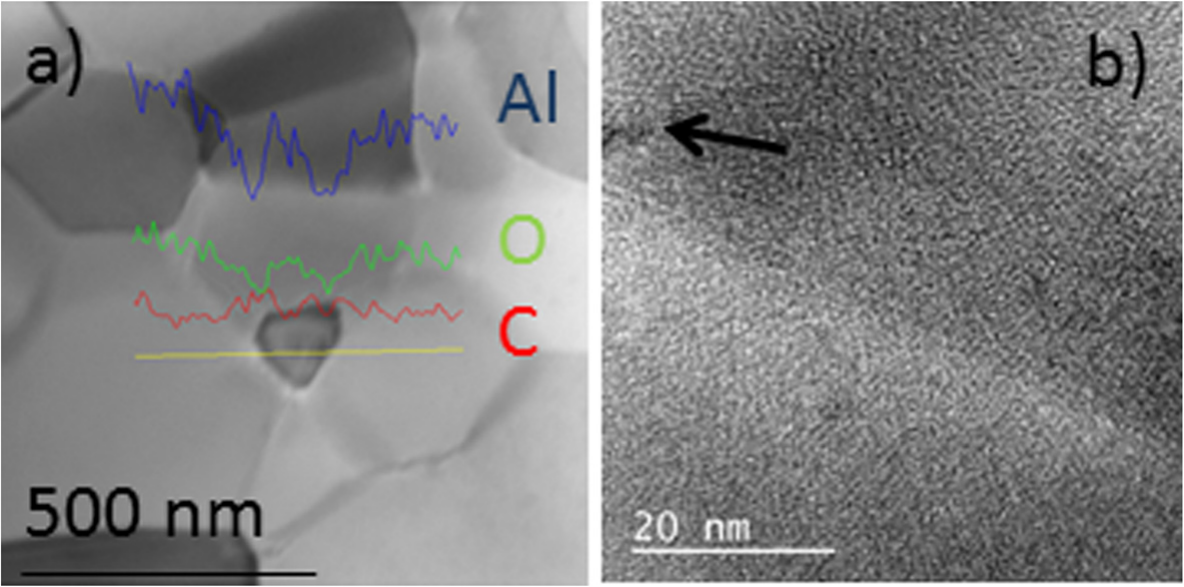
TEM images of the sample with the highest graphene content (**a**) with EDX analysis and (**b**) showing stacked graphene layers

Electron-dispersive X-ray spectroscopy performed along the yellow line in Fig. 2a on a transmission electron micrograph confirms that graphene agglomerates place on the grain boundaries and triple points. The blue and green lines indicate the presence of aluminum and oxygen, which corresponds to alumina, whereas the red line indicates the presence of carbon. It can be seen that maxima of the red line coincide with positions of minima of the blue and green lines, which happen at the alumina grain boundaries. Fig. 2b shows a high-resolution TEM micrograph in which the position of a few stacked graphene layers is indicated by an arrow.

**Fig. 3:**
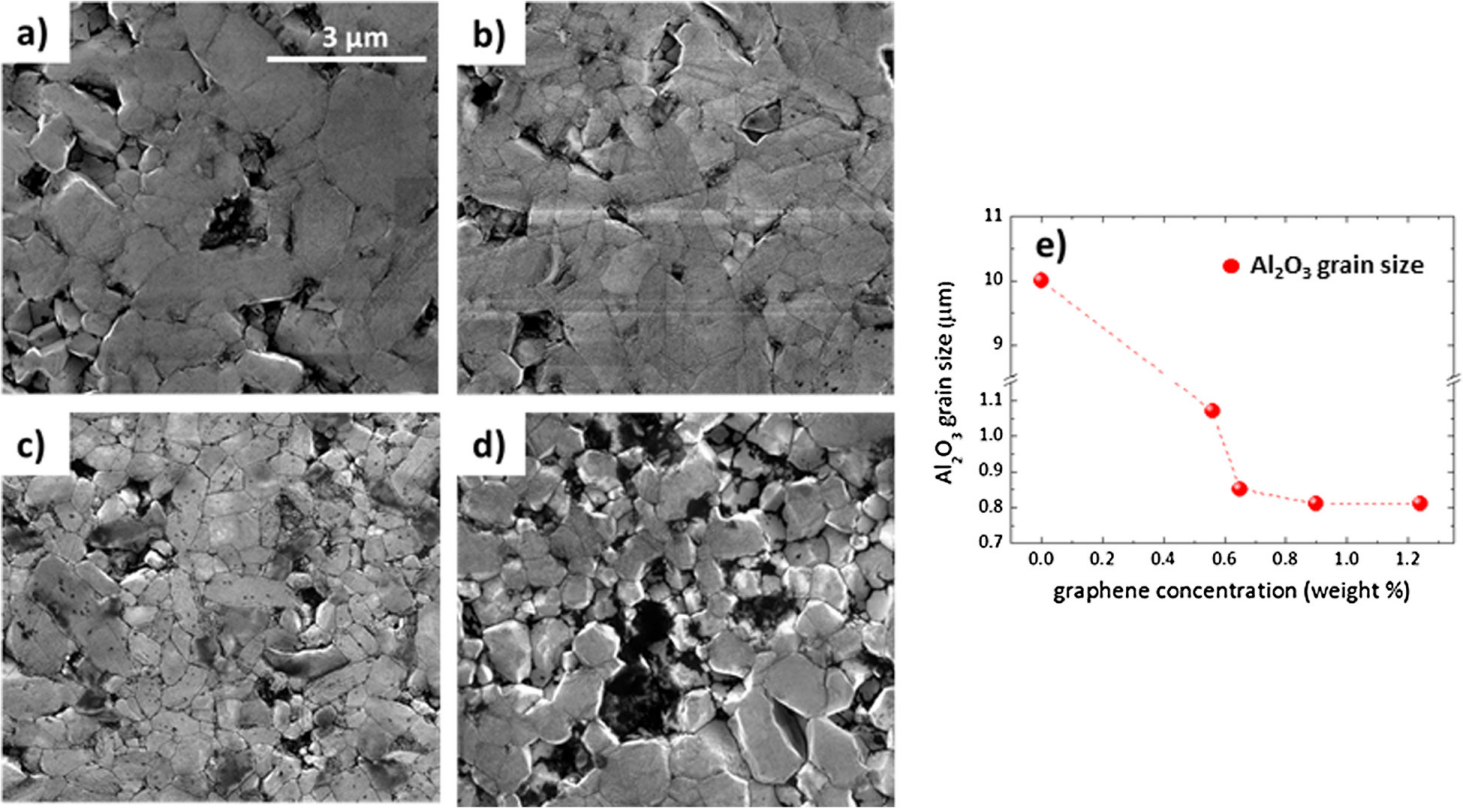
FESEM images of **a** 0.56 wt. %, **b** 0.65 wt. %, **c** 0.9 wt. %, **d** 1.24 wt. % of graphene composites and **e** alumina grain size vs. graphene weight concentration

Field emission scanning electron microscopy images (Fig. 3a–d) show differences in the Al_2_O_3_ grain sizes as a function of the graphene concentration in the samples. While monolithic alumina sintered by SPS under the same conditions presents 10-μm grain sizes [24], the graphene 0.56 wt. % sample presented an average alumina grain size around 1 μm, and while once the graphene content increases, even just to 0.65 wt. %, the alumina grain size decreases to 0.85 μm. Moreover, for larger graphene contents, the grain size shows a smooth decrease, until it reaches a lower limit around 0.8 μm. According to Fig. 3e, the grain size reduction is quite relevant, and it indicates that graphene nanosheets act as pinning centers hindering the Al_2_O_3_ grain growth. This result is analogous to the one reported in ref. [24] for alumina–carbon nanofiber composites in which the dielectric properties of the composites were shown to be strongly determined by their topology.

**Fig. 4:**
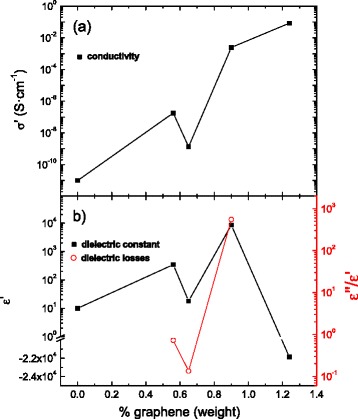
Real part of **a** conductivity and **b** dielectric constant (real part, *left*; loss tangent, *right*) of Al_2_O_3_/graphene composites vs. graphene weight concentration at a frequency of 1 kHz

Figure 4 shows the real part of conductivity (Fig. 4a), dielectric constant (Fig. 4b left), and the loss tangent (Fig. 4b right) of Al_2_O_3_/graphene composites at a frequency of 1 kHz. It should be noted that at low frequencies, the conductivity reaches a plateau regime, as it corresponds to a d.c. regime. In the case of monolithic alumina, its relative dielectric constant and conductivity are respectively *ε*′ = 10 and *σ* = 10^−11^ S·cm^−1^ in agreement with previously reported values in the literature [25, 26]. For the 0.56 % weight composite, the conductivity increases 4 orders of magnitude while the dielectric constant is multiplied by a factor of 30, which indicates the proximity of a percolation threshold. However, once the graphene content is raised up to 0.65 %, the conductivity decreases two orders of magnitude as well as the dielectric constant decreases to take values close to that of pure alumina. We have attributed this anomalous trend to the drastic changes that ceramic matrix microstructure suffers as shown in Figs. 2–3. In these micrographs, it can be seen that graphene particles are not included into alumina grains. Consequently, the available volume for the conductive phase is restricted to a small layer around the ceramic grain boundaries. In this regard, a reduction of the matrix particle size implies a larger volume to spread, so that the contact or percolation probability between graphene particles reduces in the same manner [27]. In this sense, for higher graphene concentrations (0.9 %), the conductivity and permittivity increase 8 and 4 orders of magnitude, respectively, indicating that a second percolation threshold has been reached. Higher graphene concentrations lead to minor conductivity increments whereas the dielectric constant takes negative values, which is the expected behavior of metals according to the Drude model [28]. The loss tangent shows a similar behavior to the real part of the dielectric constant.

The existence of a double percolation threshold in ceramic matrix–graphene composites can be very interesting from a technological point of view as it can be used to obtain a giant increase of the dielectric constant in a high permittivity matrix by the addition of very small amounts of graphene as will be shown next.

### BaTiO_3_/Graphene

**Fig. 5:**
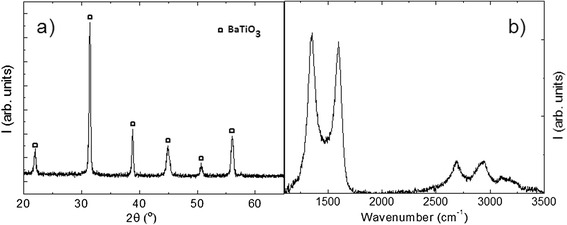
X-ray diffractogram (**a**) and Raman spectra (**b**) for the sample with the highest (0.6 % weight) graphene content

The X-ray diffraction analysis (Fig. 5a) of the sintered samples shows the diffraction peaks corresponding only to the tetragonal phase of BaTiO_3_. Again, no reaction phases are noticeable in the diffractogram corresponding to the composite with the largest amount of graphene. In this case, this is a very relevant result because some impurities, such as TiC, or different barium oxides could be expected in dense compacts if they were sintered by conventional methods. The use of SPS has allowed obtaining dense composites (theoretical density was around 100 % in all samples) by sintering them at low temperatures and for very short times. In order to study the structure of the graphene after sintering, Raman spectra were taken and are shown in Fig. 5b. Bands *D* (1355 cm^−1^) and *G* (1583 cm^−1^) are narrow and present similar intensities, with band *D* being even more intense than band *G* (*I*D/*I*G = 1.06). A qualitative comparison with the results by Botas et al. [22], considering the shapes and relative intensities of the *D*, *G*, and 2*D* bands, indicates that the state of the graphene in these composites is very similar to graphene treated at 1000 °C in the reference given above, which agrees with the sintering temperature of 1100 °C. The 2*D* peak is centered around 2690 cm^−1^ and presents a single component; therefore, according to Ferrari et al. [23], the predominant number of stacked layers is one. These results demonstrate that graphite has not been formed.

**Fig. 6:**
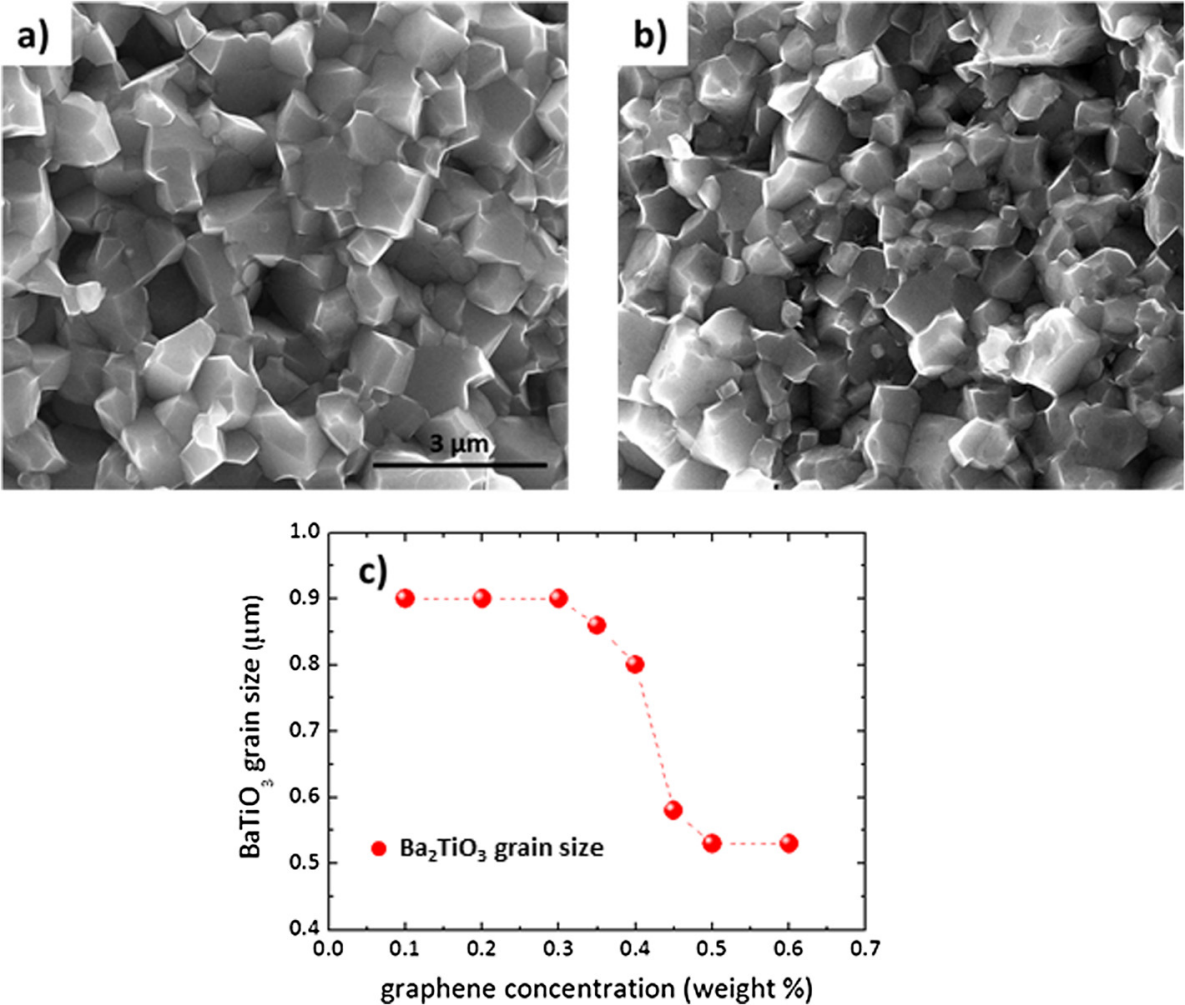
FESEM images of **a** 0.1 wt. %, **b** 0.45 wt. % of graphene composites and **c** BaTiO_3_ grain size vs. graphene weight concentration

The field emission scanning electron microscopy images of BaTiO_3_/graphene composites (Fig. 6) show a strong dependence of the average BaTiO_3_ grain sizes with the graphene concentration in the samples, similar to that shown above for Al_2_O_3_/graphene composites. In this case, composites up to 0.35 wt. % of graphene have an average BaTiO_3_ grain size of around 0.9 μm (Fig. 6a), while once the graphene content increases above 0.35 wt. %, the BaTiO_3_ grain size sharply decreases to 0.55 μm for the 0.45 wt. % composite (Fig. 6b). For larger graphene contents (>0.5 %), grain size remains invariable (Fig. 6c) around 0.5 μm. Therefore, as in the previous case, the graphene second phase acts as a pinning center hindering the BaTiO_3_ grain growth.

**Fig. 7:**
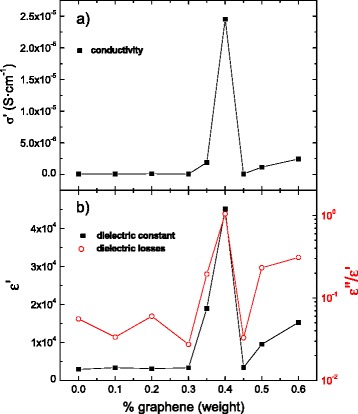
Real part of **a** conductivity and **b** dielectric constant (real part, *left*; loss tangent, *right*) of BaTiO_3_/graphene composites vs. graphene weight concentration at a frequency of 1 kHz

The dependence of the microstructure versus graphene content is expected to have a deep influence on the dielectric properties of the composites. Fig. 7 shows the real part of conductivity (Fig. 7a), dielectric constant (Fig. 7b, left), and the dielectric loss (Fig. 7b, right) of BaTiO_3_/graphene composites at a frequency of 1 kHz. Samples with graphene contents up to 0.3 % show similar conductivity values to those of pure BaTiO_3_, indicating that these composites are below the percolation threshold. In this range of graphene concentrations, the dielectric constant takes the bulk value of pure BaTiO_3_ (~2900) and the dielectric losses of the composites (~4–5 %) are due to a reduction of the Ti^4+^ to Ti^3+^ originated during the sintering process in vacuum. With increasing graphene content, the conductivity increases until it reaches a maximum value (~2.5·10^−5^ S·cm^−1^) at 0.4 % graphene. Then, conductivity abruptly decreases for the 0.45 % graphene composite, displaying again a conductivity behavior similar to that of BaTiO_3_. In a similar way as it happens in the Al_2_O_3_/graphene composites shown above, the pinning effect on the matrix grains by graphene sheets induces a local decrease (at the grain boundaries) of the effective graphene concentration and, therefore, a decrease in the composite conductivity. Finally, the conductivity of the composites increases again for concentrations over 0.45 %.

Analogously to the conductivity, the dependence of the average grain size with the graphene content has a direct influence on the real permittivity values of the composites. For this magnitude, the percolation theory predicts a non-intuitive behavior. According to the work of Efros [6], a near percolated system with conductive inclusions embedded in an insulator matrix should present a sharp maximum of the permittivity. The physical interpretation of this phenomenon can be rationalized as around the percolation threshold, conductor particles are very close between them, but completely isolated by a thin dielectric layer, so that they behave as small capacitors working in parallel; in such manner, the effective capacity is very large. Once the particle concentration exceeds the percolation threshold, particles start touching and the stored charge and capacity sharply diminish. As predicted by the percolation theory of insulator–metal systems [29, 30], a giant increase (over a factor of ten) of the dielectric constant is obtained around the two percolation thresholds. Fig. 7b (left) shows the real part of the dielectric constant of BaTiO_3_/graphene composites at a frequency of 1 kHz. In particular, the 0.4 % composite exhibits a permittivity value of ε′ ≈ 45,000 and the 0.6 % composite of ε′ ≈ 15,000 compared to the value of 2900 for the monolithic BaTiO_3_ ceramic. However, these large values of the dielectric constant are accompanied by large increases of the loss tangent (Fig. 7b, right) contrary to the results by Pecharromán et al. [31]. Given the large aspect ratio and low concentration of the conductive second phase, the control of the local microstructure becomes more difficult, which leads to both locally percolated (responsible of the large dielectric losses) and non-percolated regions.

## Conclusions

In summary, it has been shown that low concentrations of the filler produce a strong change in the microstructure of the ceramic matrix composites, particularly on the average grain size of the ceramic matrix. When the aspect ratio of the conductive filler is large enough, the pinning effect of graphene on ceramic matrix modifies the spatial distribution of the conductive phase in such a manner that the percolation threshold is reached twice as the result of the change of local graphene concentration. The dielectric properties of Al_2_O_3_/graphene and BaTiO_3_/graphene composites at low frequencies depend not only on the graphene concentration but also on the grain size and topology of the composites. This statement has been shown in two different systems, namely, Al_2_O_3_/graphene and BaTiO_3_/graphene. A notable increase (over a factor of ten) of the dielectric constant has been found in BaTiO_3_/graphene composites by introducing very small amounts of graphene. In particular, a permittivity value around 45,000 was obtained for the composite of 0.4 wt. % of graphene and around 15,000 for the 0.6 % graphene composite. This opens a route to develop giant dielectric constant composites with very low concentrations of conductive phases.
